# Response of aboveground biomass and diversity to nitrogen addition – a five-year experiment in semi-arid grassland of Inner Mongolia, China

**DOI:** 10.1038/srep31919

**Published:** 2016-08-30

**Authors:** Kejian He, Yu Qi, Yongmei Huang, Huiying Chen, Zhilu Sheng, Xia Xu, Lei Duan

**Affiliations:** 1 State Key Laboratory of Earth Surface and Resource Ecology, College of Resources Science and Technology, Beijing Normal University, Beijing 100875, P.R. China; 2 College of Resource and Environment, Yunnan Institute of Geography, Yunnan University, Kunming 650091, P.R. China; 3 Inner Mongolia Research Academy of Environmental Sciences, Hohhot 010011, P.R. China; 4 State Key Laboratory of Environmental Simulation and Pollution Control, School of Environment, Tsinghua University, Beijing 100084, P.R. China

## Abstract

Understanding the response of the plant community to increasing nitrogen (N) deposition is helpful for improving pasture management in semi-arid areas. We implemented a 5-year N addition experiment in a *Stipa krylovii* steppe of Inner Mongolia, northern China. The aboveground biomass (AGB) and species richness were measured annually. Along with the N addition levels, the species richness declined significantly, and the species composition changed noticeably. However, the total AGB did not exhibit a noticeable increase. We found that compensatory effects of the AGB occurred not only between the grasses and the forbs but also among Gramineae species. The plant responses to N addition, from the community to species level, lessened in dry years compared to wet or normal years. The N addition intensified the reduction of community productivity in dry years. Our study indicated that the compensatory effects of the AGB among the species sustained the stability of grassland productivity. However, biodiversity loss resulting from increasing N deposition might lead the semi-arid grassland ecosystem to be unsustainable, especially in dry years.

Nitrogen (N) is the primary limiting nutrient for plant growth in many terrestrial ecosystems^1^. Therefore, N fertilization has been widely used to improve soil N availability and promote plant productivity in N-limited ecosystems^2^. Because of increasing anthropogenic emissions, atmospheric N deposition has substantially increased on a global scale and is a large N source for many terrestrial ecosystems^2^,^3^,^4^. In semi-arid areas of northern China, the current rate of total N deposition is likely to be greater than 1.5 g N m^−2^ yr^−1 ^5. In addition, a higher N deposition rate could occur in the future^6^. Increasing the N input would strongly affect the plant community and would further affect ecosystem functions, such as global carbon cycles^7^,^8^,^9^,^10^.

N enrichment usually increases community productivity by stimulating plant growth^11^,^12^,^13^. However, increased N input would reduce the niche dimension, lead to long-term biodiversity loss^14^, and further decrease ecosystem functions^15^. Individual species and functional groups of the plant community respond to N enrichment differently as a result of the inherent differences of N-use efficiency and strategies^11^,^16^, causing cascading effects on competitive exclusion, species composition change and biodiversity loss^4^,^8^,^17^. Thus, a better understanding of how the productivity, biodiversity, species and community structure respond to N enrichment is essential for managing and protecting ecosystems.

Studies on N deposition and its effects on natural vegetation primarily concern temperate humid regions of the northern hemisphere^4^. In arid and semi-arid ecosystems, plant diversity and primary production are limited by both water and N^12^,^18^. These grasslands are sensitive to N enrichment because the availability of N is chronically low in these regions^12^,^19^. In these ecosystems, even small amounts of N fertilization may have important repercussions for their biodiversity and ecosystem functions^20^. Previous studies have suggested that plant responses to N addition are greater in mesic than in dry ecosystems^11^, and that the N thresholds for calcareous grasslands are higher than those for acid grasslands^21^. However, how semi-arid ecosystems respond to N enrichment remains unknown.

Drought is an extreme climatic event that occurs frequently in semi-arid zones. The frequency and severity of droughts are expected to increase in these zones^22^. Drought reduces plant cover and productivity^23^ and changes the composition of species by restraining dominant species or reducing the emergence of annual plants^24^. With the resumption of rainfall after a drought, both vegetation cover and productivity recover^23^,^24^. N addition would accelerate the productivity of aboveground tissues, resulting in increased evaporative demands, higher drought susceptibility and weakened competitive species performance^25^. Furthermore, N addition can enhance grassland recovery after a drought in arid environments^24^. However, the effect of N addition on semi-arid grassland with fluctuating precipitation has not been well demonstrated.

To understand how plant communities respond to increasing N deposition in a semi-arid ecosystem, we conducted a 5-year field experiment with six levels of N addition to simulate N deposition in a *Stipa krylovii* steppe of Inner Mongolia. The aims of this study are to investigate the response of aboveground biomass and species richness to N addition, and the interannual differences of these responses.

## Methods

### Study area

The experimental site is located at Taibus Banner in Inner Mongolia, China (115°29′10″E/42°06′44″N). The altitude is 1450 m. It is a typical semi-arid agro-pastoral ecotone between the North China Plain and the Inner Mongolia Plateau. The climate is continental and has a mean annual temperature of 1.6 °C, annual precipitation of 400 mm, and annual pan evaporation of 1900 mm. The growing season usually starts in late April and ends in late September. The temperature and precipitation were in a normal range during the years of experiment (2010–2015), except for a drought in 2011. In 2011, the precipitation during the growing season was much less than that in the other years (Table 1). The soil is a light chestnut soil (pH = 7.5). The organic carbon content and total nitrogen content are 17.44 g kg^−1^ and 1.80 g kg^−1^, respectively^26^. The annual natural N deposition is approximately 3.43 g N m^−2^ yr^−1^ 27. The experimental grassland is *Stipa krylovii* steppe, which is a typical grassland of the Eurasian steppe. Dominant species are *Stipa krylovii* and *Artemisia frigida*. The permanent experimental area (>100 ha) has been fenced with barbed wire since 1998 to exclude grazing.

### Experimental design

The N addition experiment pre-treatment was conducted on the fenced area in May 2010. The experimental site was divided into 18 plots of 3 × 6 m with additional 1-m buffer zone between the plots. The plot corners were marked with polyvinyl chloride (PVC) stakes that were approximately 1 m tall. The N addition experiment was implemented starting in 2011 with six treatments: 0 (N0), 2 (N2), 5 (N5), 10 (N10), 25 (N25), and 50 g N m^−2^ yr^−1^ (N50). The six treatments were randomly assigned to the 18 plots (three replicate plots per treatment). The NaNO_3_ solution was sprayed two times per year in early June and early July, with half of the annual amount applied each time. All plots received the same additional amount of water, which was approximately 0.5% of the annual rainfall. The N addition from well water was <0.005 g N m^−2^ yr^−1^.

### Sampling and measurements

Sampling and measurements were carried out in early September of each year, beginning in 2010. The aboveground plant material was harvested in three 0.5 m × 0.5 m quadrats for each plot. After harvest, we sorted all living plants to the following species: *Stipa krylovii*, *Artemisia frigida*, *Convolvulus ammannii*, *Cleistogenes squarrosa*, *Leymus chinensis*, and remaining species. All species were divided into two functional groups: grasses and forbs. The remaining species were almost all forbs; thus, they were classified as forbs. Samples were oven dried at 65 °C for 48 hours to constant weight. All biomass variables were converted to units of g m^−2^. Species richness was surveyed as the number of species in each plot.

### Statistical analyses

The aboveground biomass (AGB) and species richness of each plot in 2010 prior to treatment were used as a baseline against which all treatments comparisons were made. The response ratios (*R*)^28^ of the AGB and species richness were calculated to quantify the impacts of N application by comparing the variables of the after-treatment (2011–2015) to that of the pre-treatment (2010). The response ratio was calculated as


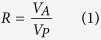


where *V*_*A*_ is the value of the variable in each plot with the N application and *V*_*P*_ is the pre-treatment value of the same variable in the same plot.

To exclude the effects caused by other factors, we revised the response ratios by the correction factor (*cf*)^29^. The *cf* was calculated as


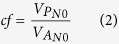


where 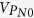 is the mean pre-treatment value of the variable in the control plot (N0) and 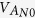 is the mean after-treatment value of the same variable in the control plot.

To perform statistical analyses, we calculated the relative effects of N addition (*E*) by transforming the revised response ratios to the natural log, which was calculated as


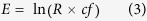


where *R* is the response ratios and *cf* is the correction factor.

Then, we calculated the relative effects of each N treatment on the total AGB, species richness, the AGB of functional groups and the common species in each N treatment year. The relative effects would be zero, positive or negative if no change, increased or decreased effects occurred with the N addition, respectively.

The relative effects of N addition on these variables were tested in a repeated-measure analysis of variance (ANOVA). The year was treated as a repeated effect. N treatments entered the model as class variables, and they were defined as continuous variables. The Student-Newman-Keuls (SNK) *post-hoc* test was performed to evaluate the differences in the relative effects at different N treatments and among experimental years for each variable. Least-squares regression line was applied in describing the relationship between the relative effects and nitrogen loads. All analyses were carried out using the statistical software SPSS21.0 (IBM Company, Armonk, NY, USA).

## Results

### Basic community characteristics in pre-treatment year

**Figure 1:**
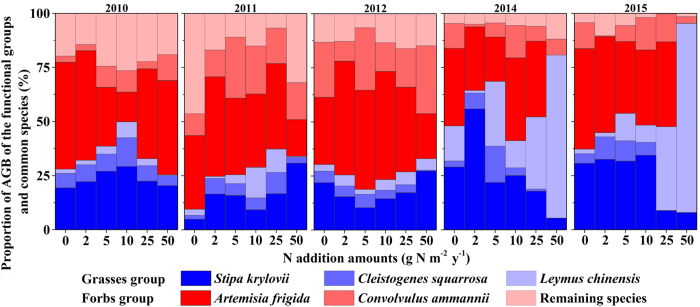


The total AGB of the treatments ranged from 90.28 to 132.66 g m^−2^ in 2010, before the experiment was implemented (Table 2). Perennial grasses and forbs accounted for 34% and 65% of the total AGB, respectively, whereas the annuals accounted for less than 1%. Thirty-three species presented in the experimental site. The species richness of the treatments ranged from 5.80 to 6.73. The common species were *Stipa krylovii*, *Artemisia frigida*, *Convolvulus ammannii*, *Cleistogenes squarrosa* and *Leymus chinensis*. These common species accounted for almost 75% of the total AGB of the community (Fig. 1). There were no significant differences in the total AGB, the AGB of the functional groups, the AGB of common species and the species richness among the treatments in 2010 (Table 2).

### Response of species composition to nitrogen addition

The species composition changed noticeably after three years of N addition. In 2014 and 2015, along with N addition levels, the proportion of grasses increased whereas that of forbs decreased; the proportion of *Leymus chinensis* increased whereas that of *Stipa krylovii* and *Artemisia frigida* decreased. In particular, *Leymus chinensis* became one of the most dominant species in the high N treatments (Fig. 1).

### Effects of nitrogen addition on the total AGB and species richness

**Figure 2:**
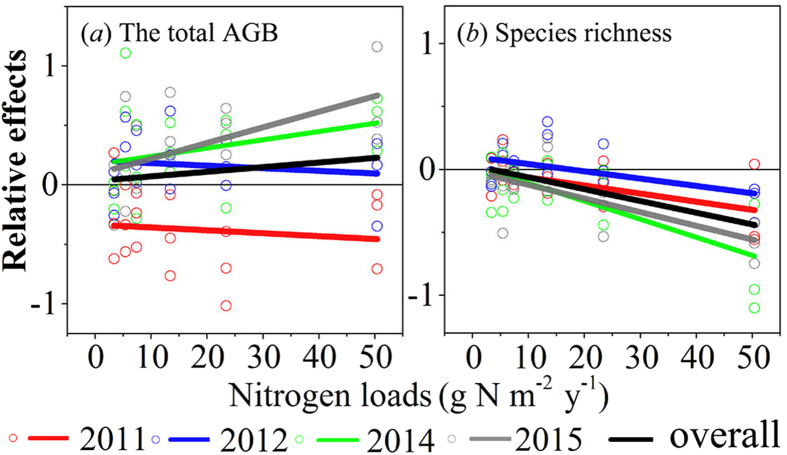
Relative effects of N addition on the total AGB (**a**) and species richness (**b**). Horizontal lines indicate neutral effects. Fitted lines are based on the least-squares regression (Supplementary Table S1).

The total AGB did not have a noticeable increase in the N addition experiment, as indicated by the almost neutral overall relative effects (Fig. 2a). The repeated measures ANOVA analysis also showed that the N addition had no significant effects on the total AGB (*F* = 1.811, *p* = 0.185, Table 3). However, the year had a significant effect on the total AGB (*F* = 19.994, *p* < 0.05, Table 3). N addition had an obvious negative effect on the total AGB in 2011. However, the relative effects on the total AGB were positive in other experimental years (Fig. 2a).

There was an obvious decrease in species richness with N addition in our experiment, as indicated by the negative relative effects on species richness both in the whole experimental period and in individual years (Fig. 2b). Furthermore, the N addition and the year had significant effects on the species richness (*F* = 6.040, *p* = 0.005; *F* = 6.235, *p* = 0.007; respectively, Table 3). The species richness decreased with increasing N addition and experimental time (Fig. 2b).

### Effects of nitrogen addition on the AGB of the functional groups

**Figure 3:**
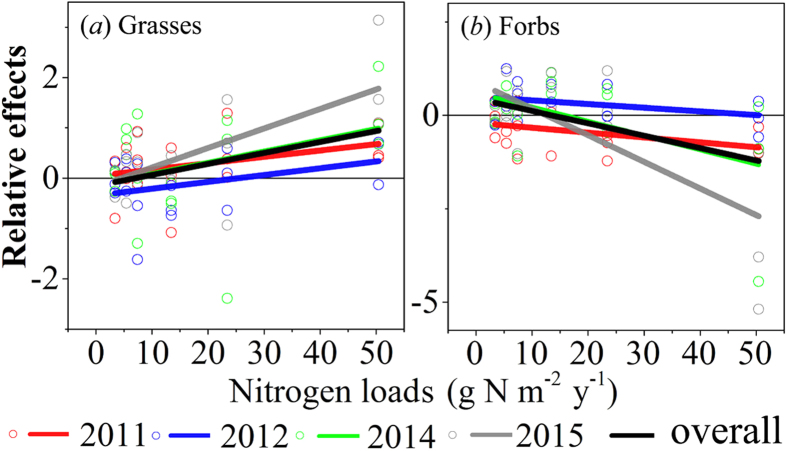
Relative effects of N addition on the AGB of grasses (**a**) and forbs (**b**). Horizontal lines indicate neutral effects. Fitted lines are based on the least-squares regression (Supplementary Table S1).

The grasses and the forbs had opposite responses to N addition. The relative effects of N addition on the AGB of grasses were negative in the lower N treatments (N 2, N5 and N10) and positive in the higher N treatments (N 25 and N 50) overall (Fig. 3a). In contrast, the relative effects on the AGB of forbs were positive in the lower N treatments and negative in the higher N treatment overall (Fig. 3b).

The results of the repeated measures ANOVA showed that the N addition had no significant effects on the AGB of grasses (*F* = 1.757, *p* = 0.196, Table 3). However, the year had a significant effect on the biomass of grasses (*F* = 4.009, *p* = 0.015, Table 3). The AGB of grasses increased significantly with N addition over the experimental time (Fig. 3a). The N addition, the year, and the interaction of N×Year had significant effects on the AGB of forbs (*F* = 3.278, *p* = 0.043; *F* = 5.808, *p* = 0.002; *F* = 3.609, *p* = 0.001; respectively, Table 3). The relative effects on the AGB of forbs increased with increasing N application and experimental time (Fig. 3b). More notably, the AGB of forbs decreased obviously in 2011, as indicated by a large negative effect. However, it had recovered to pre-treatment levels in 2012, as indicated by a positive effect (Fig. 3b).

### Effects of nitrogen addition on the AGB of common species

**Figure 4:**
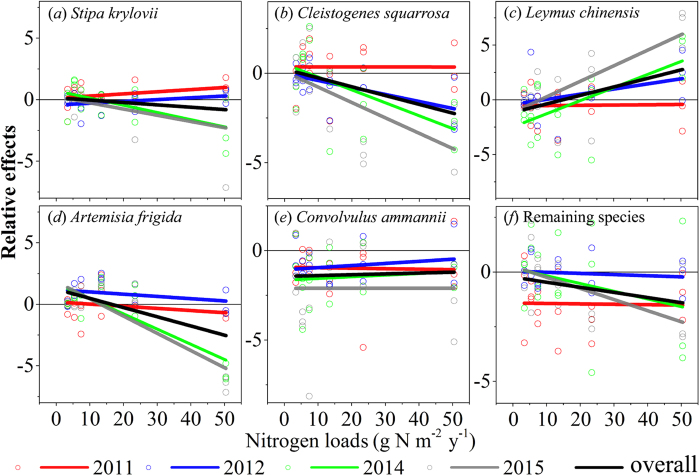
Relative effects of N addition on the AGB of the common species. Horizontal lines indicate neutral effects. Fitted lines are based on the least-squares regression (Supplementary Table S1).

Species showed different overall responses to N addition in the experiment. The relative effect on AGB of *Leymus chinensis* was positive and increased more in the higher N treatments (Fig. 4c). It was positive in the lower N treatments (N2, N5, and N10) and negative in the higher N treatments (N25 and N50) for *Stipa krylovii* and *Artemisia frigid* (Fig. 4a,d). It was negative and declined more in the higher N treatments for *Cleistogenes squarrosa* and the remaining species (Fig. 4b,f). It was negative and unchanged among the six N treatments for *Convolvulus ammannii* (Fig. 4e). More notably, there was a clear compensatory effect of biomass among Gramineae species. Along with the N addition levels, the biomass of *Stipa krylovii* and *Cleistogenes squarrosa* decreased whereas that of *Leymus chinensis* increased (Fig. 4a–c).

Species responses to N addition were lessened in 2011 compared to the other experimental years. In the year 2011, the response differences were minimal among the six N treatments for all species, as indicated by a slope of almost zero. However, the N addition enhanced the response of species in the other years because the response differences increased with increasing N application and experimental time (Fig. 4a–f).

## Discussion

### Response of the total AGB to N addition from community to species

The total AGB of the grassland did not change significantly in the N addition experiment. N addition generally have a positive effect on grassland productivity^11^,^30^. This could be because the N addition lessens the N limitation by increasing soil N availability and thus stimulates plant growth^31^. However, many experiments have found that few or no productivity changes occurred with N addition in the Inner Mongolian steppe^32^ and for some other grasslands^24^,^33^. The lack of grassland AGB response to N addition in our experiment might be largely attributed to the three factors that follow. First, primary production was co-limited by available water and N in semi-arid ecosystems^12^,^18^,^34^. Hence, water availability mediated the response of productivity to N addition^35^. The slope of the total AGB along with N addition was more gentle in the dry year (annual rainfall was approximately 260.10 mm in 2011) in our experiment (Fig. 2a). Though the increasing N additions enhanced the soil N availability, the limited precipitation might have restrained the productivity response to N addition in our experiment. Second, our experiment was launched in an area with a high natural N deposition of approximately 3.43 g N m^−2^ yr^−1^ 27. Previous studies have suggested that high background N deposition might weaken the effects of N addition^36^. Furthermore, there was a lower N saturation threshold of less than 10 g N m^−2^ yr^−1^ in a similar grassland of the Inner Mongolian steppe^32^. Therefore, the amount of the added N might have easily reached or exceeded the N saturation threshold in our experiment, which might have reduced the sensitivity of the vegetation to N additions^37^. Finally, there were opposite responses with respect to AGB between the grasses and the forbs (Fig. 3), which might have buffered the responses of community AGB to N addition and increased community stability^38^.

There was a compensatory effect of N addition on AGB between the grasses and the forbs in this experiment. The results are consistent with previous experiments that were conducted in the Inner Mongolian steppe; the biomass of grasses increased with N addition, whereas that of forbs decreased^32^,^39^. In addition, we found that there was a clear compensatory effect of biomass among Gramineae species, i.e., *Stipa krylovii*, *Cleistogenes squarrosa* and *Leymus chinensis*. After three years of the experiment (2014 and 2015), *Leymus chinensis* became one of the most dominant species in high N plots (Fig. 1). It indicated that *Leymus chinensis* was better able to take advantage of the increased N, thus resulting in an increased dominance of competition^40^. Additionally, the competitive advantages of the species that are consistently dominant in a low N environment were dwindling with increasing N addition^41^. This could be explained by the difference in their fine root morphology, the favorable effects of nitrogen and the subsequent growth responses resulting from their different nitrogen and water use efficiencies^39^,^42^,^43^. Species showed differential responses to N addition, which led to a reordering of species composition in this study. Similar results have been obtained from research in grassland ecosystems^8^,^44^. This may be a consequence of interspecific competition caused by interspecific differences in resource-use strategies and in N re-sorption proficiency^30^.

### Response of species richness to N addition

There was a noticeable decrease in species richness with N addition in our experiment. It has been verified in the Inner Mongolian steppe^9^,^11^,^31^,^39^ and other regions of the world^45^,^46^, that N addition causes an obvious reduction in species richness. Our results indicate that species had differential growth responses to N enrichment (Fig. 4). This might be a result of their inherent difference in N-use efficiency and strategies^11^,^16^. The differential growth responses thus cause cascading effects on competitive exclusion^8^,^17^. However, our results support the hypothesis of asymmetric competition, specifically, that the initial densities and establishment timing of competing species have large effects on the dynamics of plant competition because they lead to asymmetries in plant size and resource capture^47^. The grasses increased whereas the forbs decreased with N addition (Fig. 3) and N addition suppressed litter decomposition, especially grasses litter decomposition in our experiment (Supplementary Fig. S1). Accordingly, litter biomass increased with N addition in our experiment (Supplementary Fig. S2). The increase in grasses might result in increased litter accumulation and decreased light intensities at the ground surface in the community, thereby suppressing seed germination, inhibiting seedling establishment and increasing the mortality of small plants^39^. Moreover, the eutrophication caused by N addition simplifies habitats by decreasing their niche dimensionality, which would lead to loss of biodiversity^14^.

### Interannual differences in response to N addition

Our findings suggested that plant responses to N addition were lessened in the dry year (2011) than in wet or normal years. The results are consistent with previous studies, which suggested that the response of plants to N was dependent on the amount of rainfall, with a more noticeable response as a result of high amounts of precipitation^12^,^13^. It indicates that nitrogen and water availability is tightly linked through biogeochemical feedbacks^18^. In addition, N addition had an obvious negative effect on the total AGB in the dry year (Fig. 2a). A previous study suggested that N addition resulted in decreased soil moisture^34^ and that it exacerbated the decrease of water availability in soil as a result of drought conditions. Then, the decrease of water availability might increase NH_4_^+^ concentration and accelerate acid producing soil processes^48^,^49^. Finally, increased soil acidification suppresses plant growth and yield^50^. Accordingly, our results indicate that N addition exacerbates the impacts of moisture stress on the ecosystems in this arid area and that increasing N deposition might intensify productivity reductions that are caused by drought conditions.

Our findings suggested that the total AGB and species richness had a tendency to recover with N addition after drought conditions (2012). The results agree with previous research that drought conditions depress seed germination and plant growth and that increased N input can enhance grassland recovery after a drought^24^. Moreover, our findings indicate that the recovery may be a result of an increase of the forbs (Fig. 3b). This could be explained by the increased probabilities of a new species entering a community as a result of increased resource availability and altered disturbance regimes^51^.

## Conclusions

The differential responses of species and the compensatory effects of AGB between grasses and forbs or among Gramineae species might sustain the stability of grassland productivity in the context of increased N addition. However, biodiversity loss resulting from increasing N deposition might lead the semi-arid grassland ecosystem to be more unsustainable, especially in dry years. Long-term studies are needed to further test these findings and uncover their possible mechanisms.

## Additional Information

**How to cite this article**: He, K. *et al.* Response of aboveground biomass and diversity to nitrogen addition – a five-year experiment in semi-arid grassland of Inner Mongolia, China. *Sci. Rep.* **6**, 31919; doi: 10.1038/srep31919 (2016).

## Supplementary Material

Supplementary Information

## Figures and Tables

**Table 1:** Climate characteristics during the experiment (2010–2015).

Year	Growing season (April to September)		Annual precipitation (mm)
	Average monthly temperature (°C)	Average monthly precipitation (mm)	
2010	13.30 ± 1.30 a	67.05 ± 7.67 a	492.60
2011	13.15 ± 1.02 a	36.38 ± 3.43 b	260.10
2012	13.47 ± 0.81 a	68.20 ± 8.97 a	487.90
2014	14.00 ± 0.74 a	54.50 ± 3.21 a	371.90
2015	13.22 ± 0.81 a	60.53 ± 5.25 a	440.10

Different letters represent significant interannual differences for average monthly temperature or precipitation at *p* < 0.05, tested by one-way ANOVA (n = 6); mean ± standard error is shown.

**Table 2:** Mean ± standard error of the total aboveground biomass (AGB), species richness, the AGB of functional groups and common species before the N addition experiment (2010) assigned to the six treatments.

		N0	N2	N5	N10	N25	N50	*F*	*p*
The total AGB		126.25 ± 17.59	109.48 ± 14.74	132.66 ± 10.03	90.28 ± 11.77	128.21 ± 6.67	104.25 ± 8.49	1.826	0.175
Species richness		6.73 ± 0.59	6.40 ± 0.50	6.13 ± 0.18	5.67 ± 0.37	6.27 ± 0.18	5.80 ± 0.46	0.909	0.507
Functional groups	Grasses group	35.59 ± 3.74	39.06 ± 6.61	46.12 ± 4.66	46.28 ± 5.31	40.96 ± 9.13	28.23 ± 11.5	0.873	0.527
	Forbs group	90.66 ± 15.6	70.42 ± 19.13	86.54 ± 11.98	44.01 ± 9.34	87.25 ± 11.61	76.02 ± 10.5	1.668	0.217
Common species	*Artemisia frigida*	62.67 ± 13.95	61.48 ± 18.91	32.56 ± 2.44	12.78 ± 4.06	51.85 ± 13.01	48.31 ± 14.9	2.279	0.112
	*Stipa krylovii*	24.58 ± 7.78	26.99 ± 10.54	32.31 ± 10.21	27.1 ± 1.26	28.03 ± 5.41	22.59 ± 8.56	0.172	0.968
	*Leymus chinensis*	2.49 ± 1.18	2.58 ± 1.93	4.31 ± 1.56	6.78 ± 3.99	4.02 ± 0.46	0.49 ± 0.37	1.138	0.392
	*Convolvulus ammannii*	3.62±0.84	3.42 ± 1.11	11.61 ± 7.06	9.15 ± 3.95	4.17 ± 1.55	13.23 ± 9.93	0.678	0.648
	*Cleistogenes squarrosa*	8.52 ± 6.67	9.50 ± 2.43	9.50 ± 4.29	12.40 ± 0.86	8.91 ± 3.27	5.15 ± 3.52	0.352	0.872
	Remaining species	25.06 ± 5.81	17.52 ± 5.18	29.25 ± 3.48	24.59 ± 11.61	27.8 ± 9.75	21.14 ± 4.37	0.348	0.874

Differences between N treatments are indicated with *p*-values (one-way ANOVA, Num *df* = 5).

Note: The units of the biomass are g m^−2^.

**Table 3:** Repeated measures ANOVA for the relative effects of the total aboveground biomass (AGB), species richness, the AGB of functional groups and common species to N addition from 2011 to 2015.

		Nitrogen (Num *df* = 5)		Year (Num *df* = 3)		Nitrogen × Year (Num d*f* = 15)	
		Effect		*F*	*p*	*F*	*p*	*F*	*p*
The total AGB		1.811	0.185	19.994	0.000*	1.350	0.225
Species richness		6.040	0.005*	6.253	0.007*	1.246	0.314
Functional groups	Grasses group	1.757	0.196	4.009	0.015*	1.320	0.241
	Forbs group	3.278	0.043*	5.808	0.002*	3.609	0.001*
Common species	*Artemisia frigida*	24.804	0.000*	15.001	0.000*	10.476	0.000*
	*Stipa krylovii*	0.935	0.493	2.519	0.110	1.704	0.151
	*Leymus chinensis*	2.208	0.121	7.573	0.005*	3.721	0.008*
	*Convolvulus ammannii*	0.489	0.778	3.480	0.026*	1.738	0.087
	*Cleistogenes squarrosa*	3.244	0.044*	9.081	0.000*	3.183	0.002*
	Remaining species	0.750	0.602	4.310	0.011*	0.755	0.714

Note: *p < 0.05 indicates significant differences from repeated measures ANOVA.
