# Supplementary material for: Response of aboveground biomass and diversity to nitrogen addition – a five-year experiment in semi-arid grassland of Inner Mongolia, China
Source: Sci Rep. 2016 Aug 30;6:31919. doi: 10.1038/srep31919 (PMC5004133; doi:10.1038/srep31919)
Supplement: Supplementary Information [file srep31919-s1.pdf]

Supplementary information for

Response of aboveground biomass and diversity to nitrogen addition – a five-year experiment in semi-arid grassland of Inner Mongolia, China

Kejian He[(1),(2)], Yu Qi[(3)], Yongmei Huang[(1)*], Huiying Chen[(1)], Zhilu Sheng[(1)], Xia Xu[(1)], Lei Duan[(4)]

(1) State Key Laboratory of Earth Surface and Resource Ecology, College of Resources Science and Technology, Beijing Normal University, Beijing 100875, P.R. China

(2) College of Resource and Environment, Yunnan Institute of Geography, Yunnan University, Kunming 650091, P.R. China

(3) Inner Mongolia Research Academy of Environmental Sciences, Hohhot 010011, P.R. China

(4) State Key Laboratory of Environmental Simulation and Pollution Control, School of Environment, Tsinghua University, Beijing 100084, P.R. China

* Corresponding author: ymhuang@bnu.edu.cn, Phone: (+86) 10-5880 2348, Fax: (+86) 10-5880 2348

This file contains:

1) **Table S1** Parameters and statistics of the least-squares regression lines in Fig. 2, Fig. 3 and Fig. 4
2) **Fig. S1** Percentage of decomposed litter mass after 1 year. Mean ± standard error is shown.
3) **Fig. S2** Biomass of litter in the CK, lower N, and higher N treatments. Mean ± standard error is shown.

Table S1 Parameters and statistics of the least-squares regression lines in Fig. 2, Fig. 3 and Fig. 4

		Year	Intercept	Slope	R^2	*F*	*p*
**The total AGB**		2011	-0.335±0.114	-0.002±0.005	0.016	0.260	0.617
		2012	0.204±0.091	-0.002±0.004	0.020	0.329	0.574
		2014	0.168±0.125	0.007±0.005	0.100	1.778	0.201
		2015	0.087±0.110	0.013±0.005	0.338	8.169	0.011
		overall	0.031±0.075	0.004±0.003	0.022	1.548	0.218
**Species richness**		2011	0.002±0.062	-0.007±0.003	0.279	6.182	0.024
		2012	0.101±0.058	-0.006±0.002	0.265	5.770	0.029
		2014	0.036±0.085	-0.015±0.004	0.509	16.591	0.001
		2015	-0.014±0.069	-0.011±0.003	0.471	14.255	0.002
		overall	0.031±0.038	-0.009±0.002	0.337	35.544	0.000
**Functional groups**	Grasses	2011	0.045±0.192	0.013±0.008	0.133	2.463	0.136
		2012	-0.345±0.193	0.014±0.008	0.149	2.808	0.113
		2014	-0.137±0.346	0.023±0.015	0.131	2.409	0.140
		2015	-0.178±0.235	0.039±0.010	0.493	15.548	0.001
		overall	-0.154±0.129	0.022±0.005	0.190	16.464	0.000
	Forbs	2011	-0.200±0.180	-0.013±0.008	0.159	3.030	0.101
		2012	0.499±0.174	-0.010±0.007	0.103	1.837	0.194
		2014	0.600±0.378	-0.038±0.016	0.266	5.811	0.028
		2015	0.896±0.404	-0.072±0.017	0.528	17.877	0.001
		overall	0.449±0.169	-0.033±0.007	0.238	21.849	0.000

		Year	Intercept	Slope	R^2	*F*	*p*
**Common species**	*Artemisia frigida*	2011	0.187±0.381	-0.018±0.016	0.070	1.211	0.287
		2012	1.188±0.328	-0.019±0.014	0.102	1.813	0.197
		2014	1.691±0.510	-0.125±0.022	0.677	33.605	0.000
		2015	1.840±0.537	-0.141±0.023	0.708	38.883	0.000
		overall	1.226±0.287	-0.076±0.012	0.357	38.891	0.000
	Stipa krylovii	2011	0.117±0.286	0.018±0.012	0.117	2.110	0.166
		2012	-0.440±0.236	0.015±0.010	0.122	2.217	0.156
		2014	0.715±0.454	-0.059±0.019	0.374	9.548	0.007
		2015	0.245±0.580	-0.051±0.024	0.214	4.354	0.053
		overall	0.159±0.231	-0.019±0.010	0.054	3.977	0.050
	Leymus chinensis	2011	-0.553±0.559	0.002±0.024	0.001	0.011	0.918
		2012	-0.437±0.630	0.048±0.027	0.167	3.208	0.092
		2014	-2.494±0.864	0.121±0.036	0.408	11.023	0.004
		2015	-1.170±0.779	0.144±0.033	0.544	19.094	0.000
		overall	-1.163±0.400	0.079±0.017	0.237	21.765	0.000
	Convolvulus ammannii	2011	-0.930±0.532	-0.003±0.022	0.001	0.015	0.903
		2012	-1.073±0.357	0.012±0.015	0.038	0.624	0.441
		2014	-1.657±0.481	0.009±0.020	0.013	0.212	0.651
		2015	-2.107±0.829	0.000±0.035	0.000	0.000	1.000
		overall	-1.442±0.289	0.005±0.012	0.002	0.144	0.706
	Cleistogenes squarrosa	2011	0.352±0.433	0.000±0.018	0.000	0.000	0.995
		2012	-0.016±0.336	-0.040±0.014	0.327	7.763	0.013

	**Year**	**Intercept**	**Slope**	R^2	***F***	***p***
	2014	0.508±0.594	-0.073±0.025	0.346	8.454	0.010
	2015	0.082±0.652	-0.087±0.028	0.384	9.971	0.006
	overall	0.232±0.288	-0.050±0.012	0.194	16.856	0.000
Remaining species	2011	-1.422±0.438	-0.002±0.018	0.001	0.010	0.922
	2012	0.038±0.246	-0.005±0.010	0.016	0.256	0.620
	2014	0.182±0.715	-0.036±0.030	0.081	1.403	0.253
	2015	0.267±0.457	-0.051±0.019	0.307	7.102	0.017
	overall	-0.234±0.259	-0.024±0.011	0.062	4.651	0.034

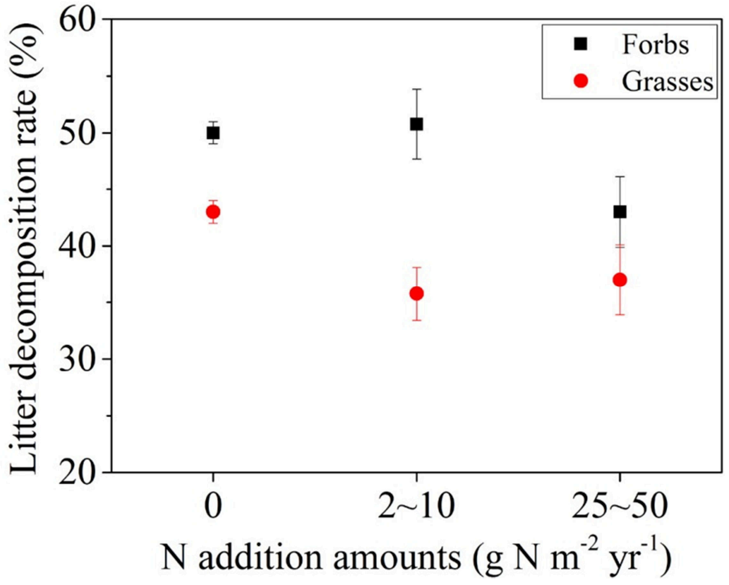

Fig. S1 Percentage of decomposed litter mass after 1 year. Mean ± standard error is shown.

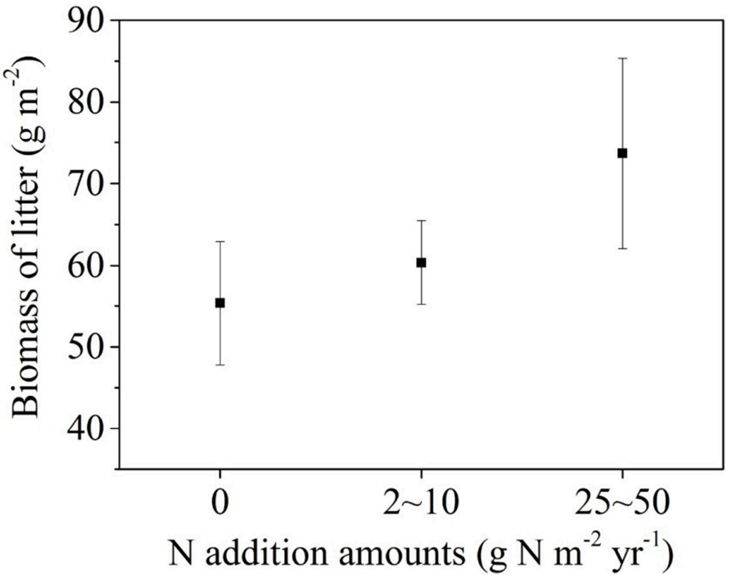

Fig. S2 Biomass of litter in the CK, lower N, and higher N treatments. Mean ± standard error is shown.
